# Combination treatment of ergosterol followed by amphotericin B induces necrotic cell death in human hepatocellular carcinoma cells

**DOI:** 10.18632/oncotarget.20285

**Published:** 2017-08-16

**Authors:** Yu-Chun Lin, Bao-Hong Lee, Jassey Alagie, Ching-Hua Su

**Affiliations:** ^1^ Department of Microbiology and Immunology, School of Medicine, College of Medicine, Taipei Medical University, Taipei, Taiwan; ^2^ Graduate Institute of Medical Sciences, College of Medicine, Taipei Medical University, Taipei, Taiwan; ^3^ Division of Hematology and Oncology, Department of Internal Medicine, Taipei Medical University Hospital, Taipei, Taiwan; ^4^ International Ph.D. Program in Medicine, College of Medicine, Taipei Medical University, Taipei, Taiwan

**Keywords:** ergosterol, amphotericin B, liver cancer, necrosis, ROS

## Abstract

The incidence of liver cancer, the second leading cause of cancer-related deaths has increased over the past few decades. Although recent treatments such as sorafenib are promising in patients with advanced hepatocellular carcinoma (HCC), the response rates remain poor thereby warranting the identification of novel therapeutic agents against liver cancer. Herein, we investigated the anti-cancer effect of ergosterol (a secondary metabolite in medicinal fungus) pretreatment followed by amphotericin B (AmB) treatment on liver cancer cell lines. We demonstrated that pretreatment with a nontoxic dose of ergosterol synergistically enhanced the cytotoxicity of AmB in both Hep3B and HepJ5 cells. The combination treatment-mediated suppression of cancer cell viability occurred through necrosis characterized by disrupted cell membrane and significant amounts of debris accumulation. In addition, we also observed a concomitant increase in reactive oxygen species (ROS) and LC3-II levels in HepJ5 cells treated with ergosterol and AmB. Our results suggest that ergosterol-AmB combination treatment effectively induced necrotic cell death in cancer cells, and deserves further evaluation for development as an anti-cancer agent.

## INTRODUCTION

Liver cancer is the second leading cause of cancer-related deaths worldwide [1]. According to the epidemiological data in the United States, there is a substantial increase in HCC mortality and incidence in the past few decades [2]. Due to the shortage of liver donors and advanced tumor stage, or liver dysfunction, only a minority of HCC patients are eligible for curative treatments, including liver resection, transplantation, and local ablation [3]. While most intermediate cases are subjected to chemoembolization, advanced cases are mainly subjected to targeted therapies such as sorafenib treatment [4]. However, the response rates to these treatments remain poor, partly because HCC often accompanies liver cirrhosis, genetic heterogeneity, and cancer drug resistance [5–7]. Therefore, there is an urgent need for the developing of complementary or alternative treatment strategies to improve the clinical outcome of conventional therapy in patients with advanced HCC.

Ergosterol is a bioactive compound widely found in medicinal fungi, such as *Cordyceps sinensis*, *Antrodia cinnamomea* and *Ganoderma lingzhi* [8–10]. It possesses various therapeutic activities including anti-inflammatory and anti-tumor effects [11, 12]. Ergosterol has been reported to reverse multidrug resistance in SGC7901/Adr cells through inhibiting the transcription of MDR1 gene and down-regulating the expression of P-glycoprotein [13]. Moreover, ergosterol inhibits breast cancer growth *in vitro* and *in vivo* by upregulating multiple tumor suppressors [12]. As a well-known polyene macrolide antifungal agent widely used in the treatment of systemic fungal infection, AmB has recently attracted wide attention due to its potential to increase therapeutic ratio of chemotherapeutic agents and reverse cancer chemotherapeutic resistance [14–17]. Apart from ergosterol sequestration and multimeric pores formation in the fungal cytoplasmic membrane leading to apoptosis, AmB also induces oxidative damage and membrane disruption [18, 19]. However, the use of AmB is associated with dose-limiting hepatic and renal toxicities [20]. Previous studies indicate that brief treatment with liposomes containing ergosterol can sensitize L1210 murine leukemia cells to the subsequent action of AmB [21]. Moreover, pretreatment with an ethanolic extract of *Antrodia camphorata* (TCEE) synergistically enhances the cytotoxic effects of AmB in human cancer cells both *in vitro* and *in vivo* [22, 23]. Since the increased susceptibility of plasma membrane to AmB was thought to be related to sterol composition and the insertion of ergostane triterpenoids from TCEE [22, 24], we speculate that ergosterol might play key a role in enhancing the anti-cancer effect of AmB.

The aim of this study was to evaluate the combined drug effect of ergosterol and AmB on human HCC cells. We demonstrated that combination treatment with ergosterol followed by AmB in a sequential manner led to a significant decrease in the viability of HCC cells in a dose-dependent manner. Significant amounts of cellular debris and autophagosome aggregation accompanied by disrupted membrane were found in cells treated with ergosterol and AmB. Furthermore, increased ROS levels and LC3-II activation were observed in HepJ5 cells treated with ergosterol and AmB. Interestingly, no significant cancer cell death was observed when either drug is used alone. These results suggest that pretreatment of ergosterol enhanced the cancer cell membrane destruction induced by AmB and provide evidence for the potential use of the combination for the treatment of liver cancer.

## RESULTS

**Figure 1:**
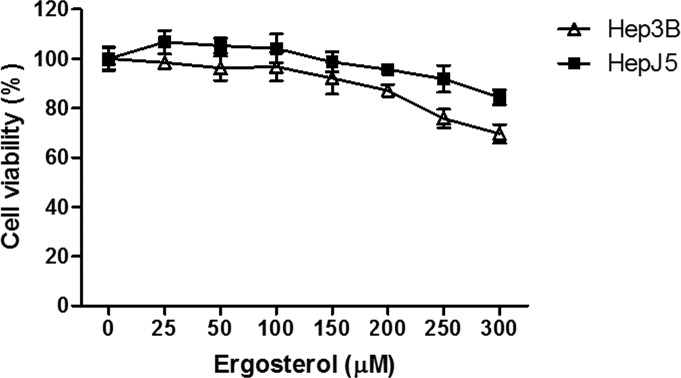
Ergosterol (300 μM) slightly inhibited cancer cell growth at the highest concentration HCC cells, Hep3B and HepJ5 were treated with ergosterol for 48 hours before analyzing cell viability. Data represents the mean ± SD of three independent experiments.

**Figure 2:**
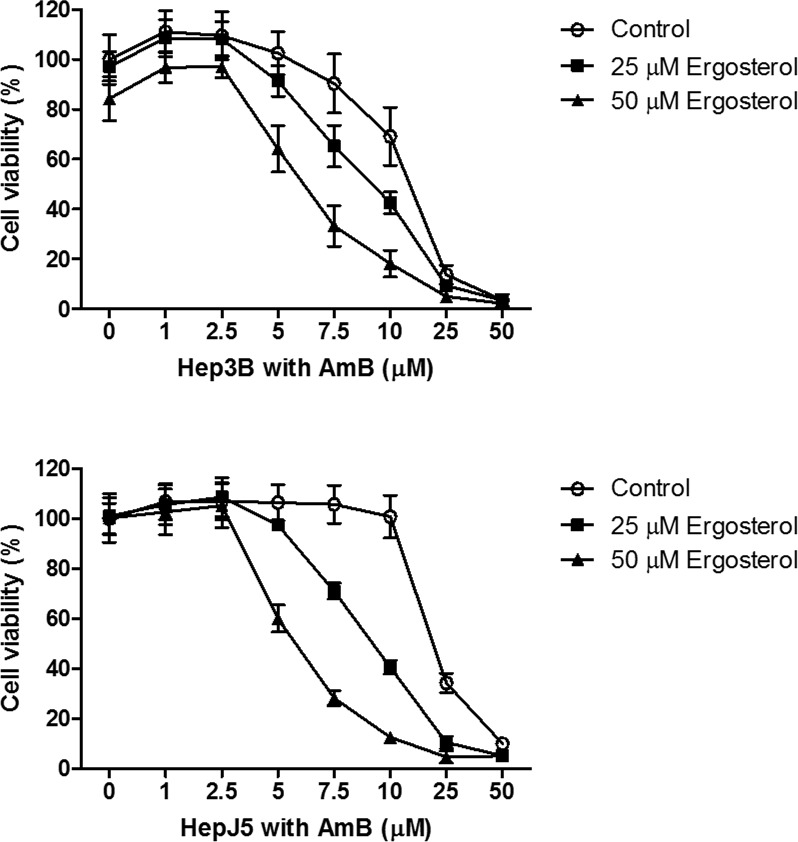
Ergosterol pretreatment potentiated the cytotoxicity of AmB in Hep3B and HepJ5 cells Cells were first pretreated with 0 to 50 μM ergosterol for 24 hours, followed by treatment with 0 to 50 μM AmB for an additional 24 hours, and the cell viability was determined by crystal violet staining. Data represents the mean ± SD of three independent experiments.

To evaluate the antitumor potential of ergosterol on HCC cells, Hep3B and HepJ5 cells were treated with 0 to 300 μM ergosterol for 48 hours and cell viability was analyzed by crystal violet staining. As depicted in Figure 1, at the highest concentration, ergosterol induced minimal toxicity on both Hep3B and HepJ5 cells. To investigate the combined drug effect of ergosterol with AmB, Hep3B and HepJ5 cells were pretreated with 0 to 50 μM ergosterol for 24 hours followed by 0 to 50 μM AmB treatments for an additional 24 hours. Pretreatment with ergosterol dramatically enhanced the cytotoxicity of AmB (Figure 2). The half-maximal inhibitory concentration (IC_50_) analysis indicates that compared with single treatment of AmB, combination of ergosterol and AmB reduced the IC_50_ values of Hep3B and HepJ5 cells from 14.54 to 6.66 and 18.65 to 4.07, respectively (Table 1). The ergosterol and AmB combination drug effect was further analyzed by the Chou-Talalay method to obtain the combination index (CI) (Table 2) which allows quantitative determination of drug interactions. The CI suggested that ergosterol and AmB (5 to 25 μM) had a synergistic effect on Hep3B and HepJ5. AmB only was more effective in suppressing cell growth on Hep3B than HepJ5 cells. Intriguingly, the combined effect of ergosterol and AmB on Hep3B cells was relatively moderate compared to HepJ5 cells. These data all together, suggest that HepJ5 cells are more resistant to either ergosterol or AmB treatment alone but more susceptible to ergosterol pretreatment combined with AmB.

**Table 1:** IC_50_ analysis on Hep3B and HepJ5 cells pretreated with ergosterol for 24 hours, followed by treatment with AmB for an additional 24 hours. The IC_50_ values of AmB were analyzed based on cell viability data obtained from Figure 2 by using the CalcuSyn software

		Ergosterol (μM)		
		0	25	50
AmB (μM)	Hep3B	14.54	10.64	6.66
	HepJ5	18.65	12.72	4.07

**Table 2:** CI analysis on Hep3B and HepJ5 cells pretreated with ergosterol for 24 hours, followed by treatment with AmB for an additional 24 hours. The CI values were analyzed based on cell viability data obtained from Figure 1 and 2 by using the CalcuSyn software. CI < 1 indicates a synergistic effect, CI = 1 indicates an additive effect, and CI > 1 indicates an antagonistic effect

		Combination index			
		Hep3B Ergosterol (μM)		HepJ5 Ergosterol (μM)	
		25	50	25	50
	5	0.94	0.57	0.76	0.36
	7.5	0.72	0.45	0.48	0.32
AmB (μM)	10	0.67	0.44	0.42	0.30
	25	0.81	0.64	0.58	0.47
	50	1.09	0.93	0.90	0.92

**Figure 3:**
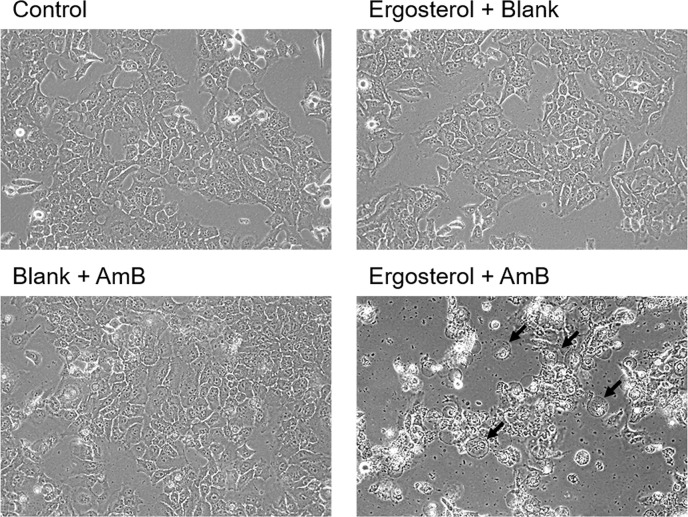
Ergosterol followed by AmB treatment induced cell rounding and debris accumulation in HepJ5 cells HepJ5 cells were first pretreated with 25 μM ergosterol for 24 hours, followed by treatment with 10 μM AmB for an additional 6 hours, and morphological observation was performed with a light microscope. Rounded cells are indicated by arrows. Magnification = 200x.

AmB was previously reported to induce membrane pore formation and alter cell membrane permeability, resulting in cell swelling, rounding and lysis [25, 26]. Therefore, we examined whether combined treatment of ergosterol and AmB could induce alterations in cell morphology. HepJ5 cells were treated with ergosterol followed by AmB and the morphology of cells were microscopically observed. In agreement with the reference cited above, our results demonstrate large amounts of rounded cells and debris in HepJ5 cells (Figure 3).

**Figure 4:**
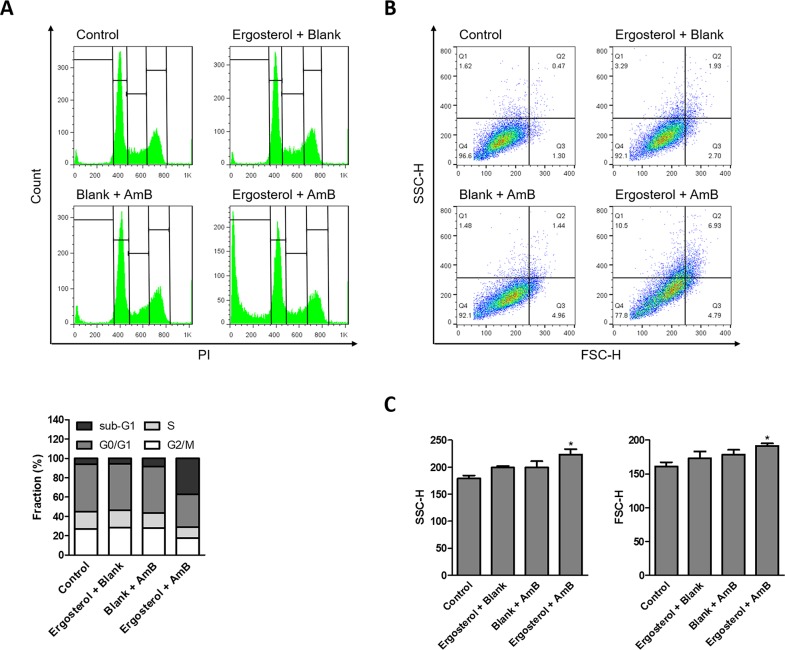
Ergosterol followed by AmB treatment increased sub-G1 population in HepJ5 cells Flow cytometry analysis of HepJ5 cell cycle after pretreatment with ergosterol for 24 hours, followed by treatment with AmB for an additional 6 hours. **(A)** Representative data are shown. Quantitative analysis of different cell phase populations is reported as mean percentages of three independent experiments in triplicates. **(B)** Live cells were gated and the internal complexity and cell size are depicted by SSC and FSC, respectively. Data are representative of three experiments. **(C)** Mean intensities of SSC and FSC quantified in ergosterol and AmB treated cells. Data represents the mean ± SD of three experiments.

**Figure 5:**
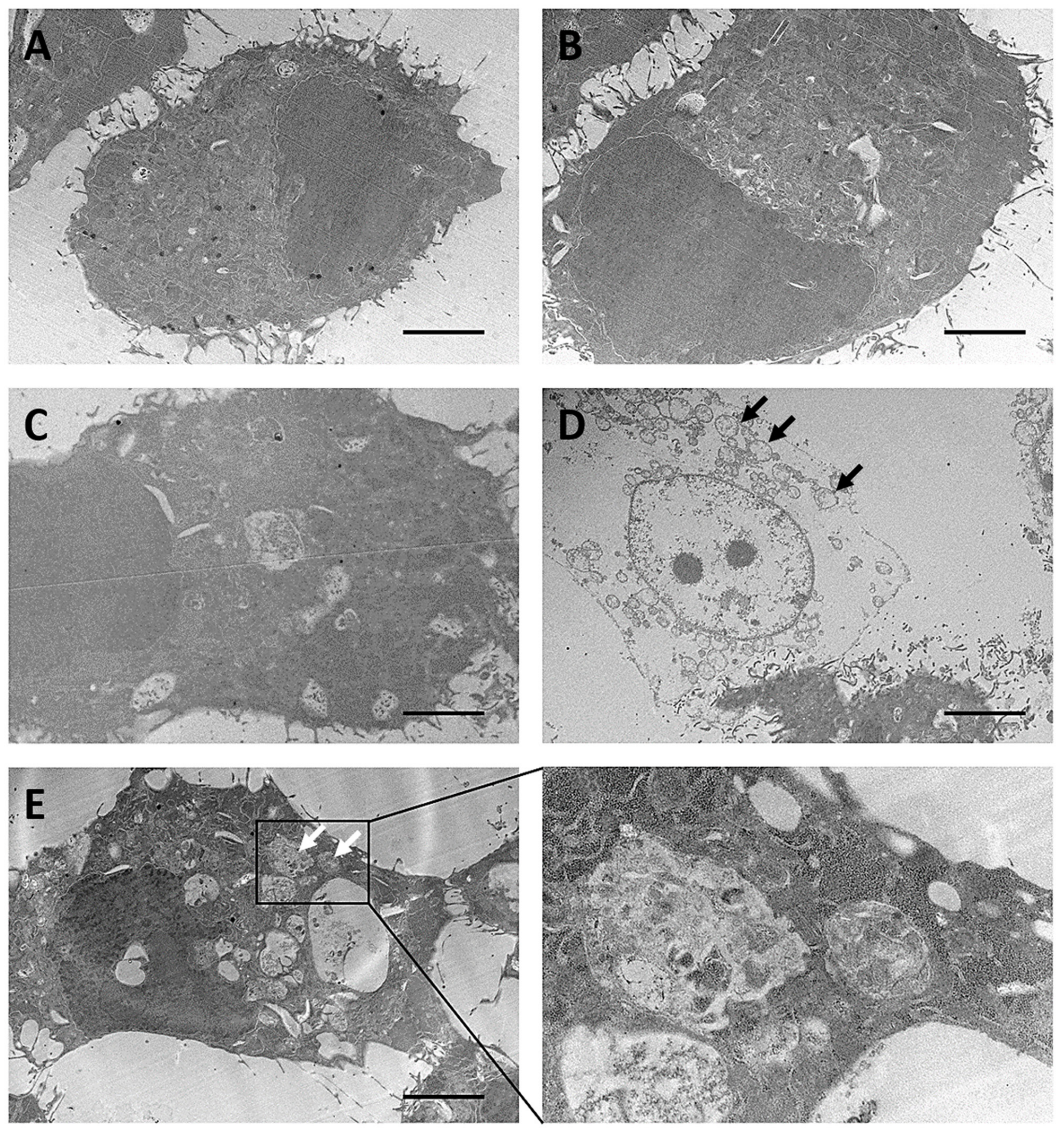
Ultrastructural analysis of ergosterol followed by AmB treatment of HepJ5 cells HepJ5 cells were first pretreated with ergosterol for 18 hours, followed by treatment with AmB for an additional 3 hours. **(A)** Control. **(B)** Cells treated with ergosterol. **(C)** Cells treated with AmB. **(D & E)** Cells treated with ergosterol and AmB. Vacuole-like structures (black arrows) and autophagosome-like double-membrane vesicular structures (white arrows) are shown. Data are representative of three experiments with similar results. Scale bar: 5 μm.

We next performed flow cytometry to further analyze ergosterol and AmB-induced cell rounding and cell debris accumulation. The untreated control and HepJ5 cells with single treatment of either drug had very little sub-G1 population. In contrast, The HepJ5 cells treated with ergosterol and AmB produced a large amount of sub-G1 population indicative of cell death (Figure 4A). In addition, the percentages of cells with high side scatter (SSC) and forward scatter (FSC) which respectively correlate to internal complexity and cell size were both increased (Figure 4B), as well as their associated mean intensities (Figure 4C). These results suggest that apart from cell debris accumulation, the combined treatment of ergosterol and AmB increases the internal cellular complexity and cell size. To determine the ultrastructural changes in HepJ5 cells induced by ergosterol and AmB treatment, the cells with or without the combined treatment were examined by transmission electron microscopy (TEM). Untreated controls and treated HepJ5 cells with either drug alone showed no ultrastructural differences (Figure 5A-5C). In contrast, combined treatment of HepJ5 with ergosterol and AmB induced cell membrane disruption and the formation of vacuole-like structures as demonstrated in Figure 5D. The autophagosome-like double-membrane vesicular structures are depicted in Figure 5E. Together, these results suggest that the combined drug treatment induces cell death by disrupting cell membrane integrity.

**Figure 6:**
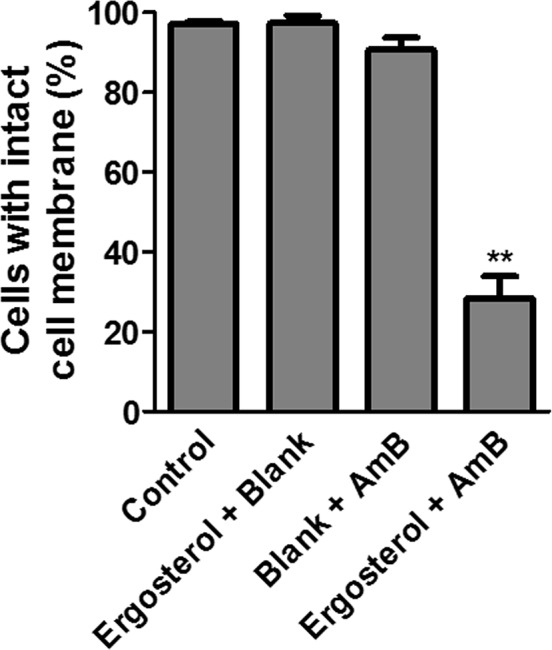
Combined treatment of ergosterol and AmB disrupted cell membrane integrity of HepJ5 cells Dye uptake assay of HepJ5 cells pretreated with ergosterol for 24 hours, followed by treatment with AmB for an additional 2 hours. Data represents the mean ± SD of three experiments.

To further investigate the mechanism of cell death, dye uptake assay was performed to determine cell membrane integrity, which should be lost if cells die of necrosis [27]. At the end of the treatment with ergosterol and AmB, the cells were stained with trypan blue; a dye that enters into cells only when cell membrane integrity is compromised [28]. There was a significant amount of dye uptake in the combined treatment group as compared to either drug treatment alone (Figure 6). Our results therefore suggest that the antitumor activity of ergosterol pretreatment combined with AmB could satisfactorily be explained by its membrane-disruptive activity leading to necrosis.

**Figure 7:**
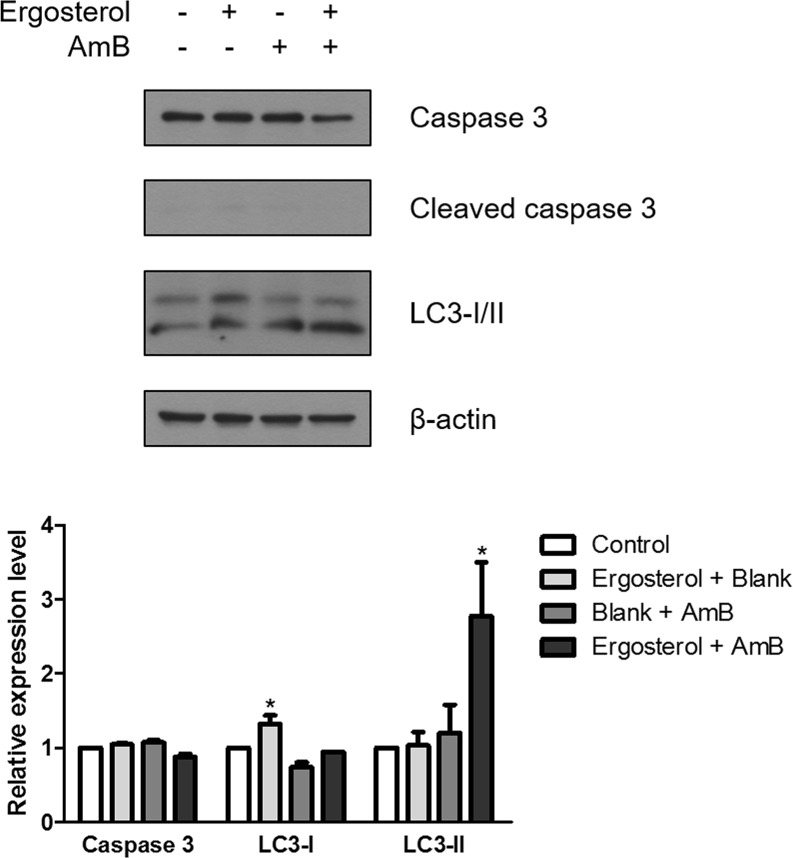
Ergosterol combined with AmB upregulated LC3-II expression in HepJ5 cells Cell lysates prepared from HepJ5 cells pretreated with ergosterol for 24 hours, followed by treatment with AmB for 1 hour were subjected to Western blot analysis for expression of caspase 3 and LC3-I/II. Quantification of the Western blot by densitometry analysis is shown below. Data represents the mean ± SD of three experiments.

As sub-G1 populations are indicative of cell death, in order to discern the involvement of other forms of cell death besides necrosis, the expression levels of caspase 3 and LC3 were examined by western blot analysis [29]. Results showed no alteration in the level of cleaved caspase 3. In contrast, ergosterol pretreatment with AmB induced a significant upregulation of the autophagosomal marker LC3-II indicating the stimulation of the autophagic process (Figure 7).

**Figure 8:**
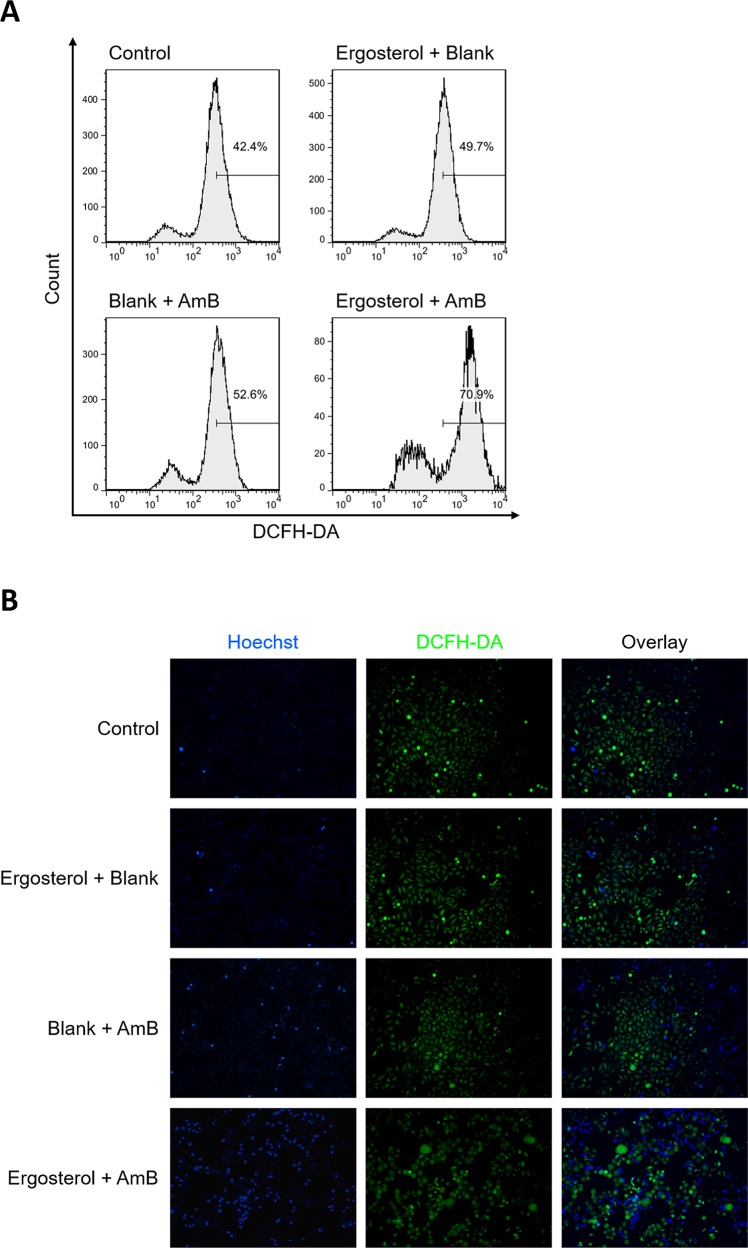
Combined treatment of ergosterol and AmB stimulated ROS production in HepJ5 cells **(A)** HepJ5 cells were pretreated with ergosterol for 24 hours, followed by treatment with AmB for 1 hour, and subsequently stained with DCFH-DA, and immediately subjected to flow cytometry analysis. **(B)** Fluorescence microscopic images of intracellular ROS probed by DCFH-DA. Data are representative of three experiments.

AmB has been shown to induce oxidative stress in cells [18]. Therefore, we further examined the potential effect of ergosterol pretreatment combined with AmB on necrotic cell death in HCC cells by measuring cellular ROS production. Cellular ROS was quantified using flow cytometry with DCFH-DA, a non-fluorescent dye emitting green fluorescent after cellular oxidation [30]. As compared to the untreated controls, treated cells with either drug alone induced only slightly elevated levels of ROS. Conversely, ergosterol pretreatment combined with AmB significantly enhanced the cellular ROS levels in HepJ5 cells as depicted by the rightward-shift in fluorescence (Figure 8A). In order to directly observe ROS generation, HepJ5 cells were stained with DCFH-DA and analyzed by epifluorescence microscopy. In contrast to either drug treatment alone, the combined treatment increased the number of green fluorescent cells which is indicative of ROS production (Figure 8B). The results above illustrated that ergosterol pretreatment combined with AmB induced a significant increase in intracellular ROS levels.

## DISCUSSION

Ergosterol is the primary sterol of fungi and has been found to possess antitumor properties in human melanoma, glioblastoma, colon, ovarian, lung, and breast cancer cells [12, 31, 32]. In these studies, the concentrations of ergosterol used exhibited little effect on the tested HCC cells, Hep3B and HepJ5. Due to the overexpression of surviving and GRP-78, HepJ5 cells were shown to be more resistant to TCEE and some chemotherapy agents [33]. Our results support the previous finding that HepJ5 cells exhibit a greater degree of drug resistance with a higher AmB IC_50_.

Since ergosterol is suggested to be the main anti-cancer ingredient in medicinal fungi and responsible for the high sensitivity of the fungal membrane to AmB [22, 34], it is worthy to clarify the combination effects of ergosterol followed by AmB on liver cancer cell lines. In the present study, we found that pretreatment with nontoxic dose of ergosterol further enhanced the tumor suppression efficiency of AmB on Hep3B and HepJ5 cells, and this enhancement was shown to be a synergistic effect between ergosterol and AmB by the results of CI analysis. These results suggest that ergosterol pretreatment can potentiate AmB-induced cancer cell death at lower doses thereby, minimizing the adverse effects associated with high AmB dose.

It is important to note that the ergosterol-enhanced AmB tumor suppression efficiency varies between Hep3B and HepJ5 cells. In fact, the combination treatment with ergosterol and AmB in a sequential manner is more effective in both IC_50_ reduction and CI analysis on HepJ5 cells. These results indicate that the aforementioned dosing regimen may exert a more potent anti-cancer effect on HepJ5 cells which are more resistant to either drug alone. This finding will be helpful to predict the drug response of combination treatment with ergosterol and AmB on liver cancer patients.

The well-known mechanisms of the action of AmB are pore-forming activity and oxidative damage leading to apoptosis and necrosis [18, 35]. In our study, we found that small pieces of debris were produced in the cultures of HepJ5 cells treated with ergosterol followed by AmB, and internal complexity as well as cell size were both increased. Our results showed that the drug combination disrupts the integrity of the cell membrane leading to intracellular vacuolization that could potentially trigger necrosis. Because autophagy targets damaged organelles for lysosomal degradation, the AmB-ergosterol-induced damage may lead to the activation of the autophagic process [36].

Furthermore, we explored the molecular mechanism underlying this effect. Our data showed that intracellular levels of ROS and LC3-II were upregulated in ergosterol-AmB combination treated HepJ5 cells. It has been identified that AmB mediates cell death through the significant increase of intracellular ROS production [37, 38]. ROS-mediated modification of autophagic proteins leads to the accumulation of LC3-II, thus allowing autophagosome to be correctly elongated [39]. Accumulating autophagosomes can become toxic and trigger necrosis [40]. Thus, we speculated that autophagy was induced without autophagosome maturation. In addition, the overproduction of ROS has been found to occur in cells undergoing necrosis [41, 42]. The induction of ROS may inhibit apoptosis and favor necrotic cell death. This is consistent with our results as we did not notice any significant upregulation of cleaved caspase 3 after ergosterol-AmB combination treatment.

**Figure 9:**
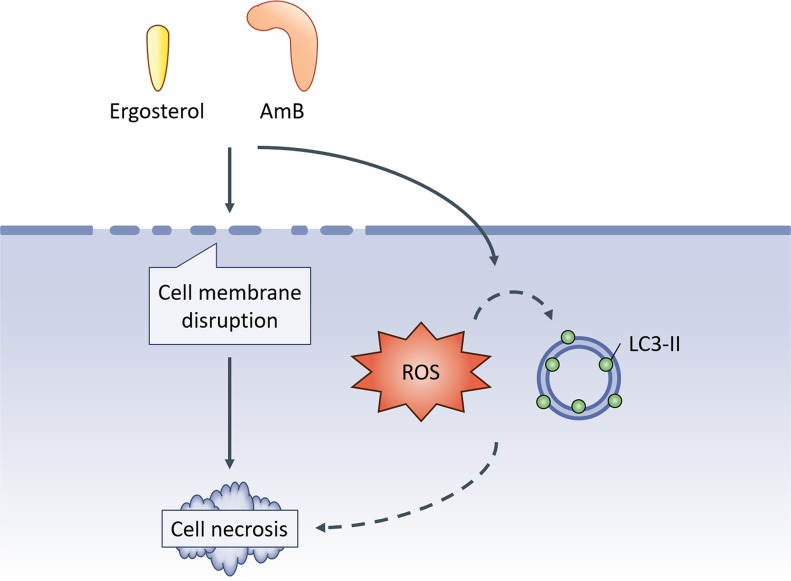
Schematics of the study Ergosterol pretreatment followed by AmB cooperatively disrupts cell membrane integrity ultimately leading to necrotic cell death as well as a concomitant increase in ROS production and LC3-II expression.Whether the induction of ROS and LC3-II contributes to ergosterol-AmB-induced cancer cell death is not clarified in this study.

In summary, we investigated the anti-cancer activity of ergosterol pretreatment followed by AmB and showed that ergosterol pretreatment synergistically enhanced the cytotoxicity of AmB in HCC cells leading to necrotic cell death (Figure 9). Our findings provide new insights into the mechanism of ergosterol-AmB-induced anti-cancer effects on liver cancer cell lines and support the potential use of ergosterol-AmB combination treatment as a novel therapeutic strategy in liver cancer patients.

## MATERIALS AND METHODS

### Chemicals

Ergosterol, AmB, Crystal violet, propidium iodide (PI), sodium dodecyl sulfate (SDS), RNase A, Triton X-100, trypsin, trypan blue, and dichloro-dihydro-fluorescein diacetate (DCFH-DA) were purchased from Sigma-Aldrich Co. (St. Louis, MO, USA). Dulbecco's modified Eagle's medium (DMEM), fetal bovine serum (FBS), penicillin, and streptomycin were purchased from Thermo Fisher Scientific (Waltham, MA, USA). The Bio-Rad protein assay dye was from Bio-Rad Laboratories (Hercules, CA, USA). The anti-caspase 3 antibody, anti-LC3-I/II antibody, anti-β-actin antibody, and anti-cleaved caspase 3 antibody were purchased from Cell Signaling Technology, Inc. (Danvers, MA, USA).

### Viability and cell death assay

Human HCC cell lines Hep3B and HepJ5 were used to test the effects of ergosterol and AmB in inducing cancer cell death. In brief, cells (5 × 10^3^ cells) were seeded in 96-well tissue culture plates in DMEM supplemented with 10% FBS, 100 U/mL Penicillin and 100 μg/mL Streptomycin at 37°C, 5% CO_2_. After overnight cell inoculation, noncytotoxic doses of ergosterol (25 and 50 μM) were first added to the cultures for 24 hours, followed by treatment with AmB for an additional 24 hours. Cell viability was analyzed by crystal violet staining. Microscopic observation was performed by Olympus CKX41 inverted microscope (Shinjuku, Tokyo, Japan) and photographed at 200x magnification before cell viability assay.

### Cell cycle analysis

HepJ5 cells were seeded as 3 × 10^5^ cells per 6 cm dish, incubated overnight, and then treated with 25 μM ergosterol for 24 hours, followed by treatment with 10 μM AmB for an additional 6 hours. After treatment, the cells were washed, trypsinized, collected, and resuspended in 1 mL phosphate buffered saline (PBS) with 4 mL 75% ethanol at −20°C overnight for cell fixation. Fixed cells were centrifuged and washed by 5 mL PBS at room temperature. Before analysis, cell suspensions were mixed with 1 mL propidium iodide buffer alone (0.2 mg/mL RNase A, 0.1% triton X-100, and 20 μg/mL PI) at room temperature for 15 min. Stained cells were finally measured by the FACScan flow cytometer (BD Biosciences, San Jose, CA, USA) and analyzed by the CellQuest software (BD Biosciences). As a control, cells treated with either ergosterol or AmB were used.

### Transmission electron microscopy

HepJ5 cells in 2-well Nunc Labtek chamber slides (Thermo Fisher Scientific) were treated with 25 μM ergosterol for 24 hours, followed by 10 μM AmB for an additional 3 hours. Cells were briefly trypsinized, pelleted, rinsed and resuspended in 2% paraformaldehyde and 2.5% glutaraldehyde fixative (Sigma-Aldrich). Cell pellets were post-fixed in osmium tetroxide, and dehydrated with an alcohol series. Samples were embedded in EPON resin and polymerized at 62°C for 48 hours. Sections were generated and placed on copper grids. Cells were examined using a Hitachi HT7700 Transmission Electron Microscope (Chiyoda, Tokyo, Japan). Electron microscopy services were performed by the Taipei Medical University Core Facility (Taipei, Taiwan).

### Trypan blue uptake assay

HepJ5 cells were seeded as 3 × 10^5^ cells per 6 cm dish, incubated overnight, and then treated with 25 μM ergosterol for 24 hours, followed by treatment with 10 μM AmB for an additional 2 hours. Cell membrane integrity of HepJ5 cells was analyzed using trypan blue. Pelleted cells were suspended in 100 μl of DMEM and 20 μl of cell suspension was mixed with equal volume of the trypan blue solution (0.4% in PBS; Sigma-Aldrich). After 5 min of incubation at room temperature, the cells were counted in a Neubauer Improved hemocytometer. The percentage of unstained cells indicating intact cell membrane was calculated.

### Western blotting

After being treated with 25 μM ergosterol for 24 hours, followed by treatment with 10 μM AmB for 1 hour, HepJ5 cells were lysed by the lysis buffer containing protease inhibitor (CALBIOCHEM, La Jolla, CA, USA) on ice for 30 min and centrifuged to obtain clear cell lysates. Protein concentrations were determined by Bio-Rad protein assay kit (BioRad Laboratories) and equalized for the sodium dodecyl sulfate polyacrylamide gel electrophoresis. Separated proteins were transferred to polyvinylidene fluoride membranes (Pall Corp., Port Washington, NY, USA), and the membranes were incubated with primary antibodies at 4°C overnight after blocking with 5% milk. The membranes were eventually washed three times, incubated with secondary antibodies for 2 hours, and washed three times again. The membranes were developed with WesternBright ECL kit (Advansta, Menlo Park, CA, USA) and visualized in the dark room. Immunoreactivity was quantified using ImageJ software (National Institutes of Health, Bethesda, MD, USA).

### ROS detection assay

ROS detection assay was performed as described [43]. In brief, cells were treated with ergosterol and AmB as described above. Cells were suspended in 500 μL of PBS, and stained with 10 μM (final concentration) of DCFH-DA followed by a 20 min incubation at 37°C. Nuclei were demarcated with Hoechst stain (Sigma-Aldrich) and ROS level was assayed by either flow cytometry or fluorescence microscopy with EVOS FL Cell Imaging System (Thermo Fisher Scientific).

### Statistical analysis

Experimental results were analyzed in triplicates and expressed as means ± standard deviation (SD). The results were subjected to statistical analysis by one-way ANOVA and Student's *t*-test. The level of significance was set at p < 0.05 and p < 0.01 respectively. The IC_50_ and the CI of ergosterol with AmB were analyzed by using the CalcuSyn software (Biosoft, Great Shelford, Cambridge, UK), which is based on Chou-Talalay median effect method [44, 45]. The obtained CI value indicates additive (=1), antagonistic (>1), or synergistic (<1) effects.
